# UP165, A Standardized Corn Leaf Extract for Improving Sleep Quality and Mood State

**DOI:** 10.1089/jmf.2021.0197

**Published:** 2023-01-12

**Authors:** Shawn M. Talbott, Julie A. Talbott, Lidia Brownell, Mesfin Yimam

**Affiliations:** ^1^ Human Clinical Trial, GLH Nutrition LLC, Draper, Utah, USA.; ^2^ Unigen, Inc., Tacoma, Washington, USA.

**Keywords:** corn leaf, cortisol, deep sleep, mood

## Abstract

UP165, a standardized *Zea mays* (corn) leaf extract, was evaluated for its effect on sleep quality and overall well-being in a double-blind, placebo-controlled clinical trial, administered orally at 250 and 500 mg/day. Participants (*n* = 45) (age range 19–73) consumed the supplement or placebo ∼60 minutes before bedtime daily for 4 weeks. Measurements that were evaluated were as follows: daily sleep quality, as monitored by a fitness tracker; the Pittsburgh Sleep Quality Index (PSQI) for sleep quality and efficiency; salivary cortisol levels for stress; and Profile of Mood States (POMS) to assess general well-being. Participants who received UP165 showed a statistically significant and dose-correlated reduction in salivary cortisol (up to 36%); an increase in deep sleep time (up to 30 minutes); increased total sleep time (up to 10%); an improvement in sleep quality (up to 49%), and an enhanced POMS (36–58%). Participants in the UP165 group showed a sevenfold increase in deep sleep time and a 10-fold increase in PSQI sleep quality improvement compared to placebo. Collectively, UP165 is a safe nutritional supplement clinically proven for a 24-hour support with better quality and efficiency of sleep at night and an improved mood state and overall well-being during the day. The clinical study has been ISRCTN registered with study ID ISRCTN68282897.

## INTRODUCTION

Current Centers for Disease Control and Prevention (CDC) data show that about 70 million U.S. adults have sleep-related problems.^1^ In a recent survey, 40% of U.S. adults reported struggling with mental health, while 31% showing depression and anxiety symptoms.^2^ Unfortunately, safe and efficacious treatment options to address poor sleep and mental wellbeing are inadequate. Although pharmaceutical drugs like benzodiazepines, antidepressants, and anxiolytics are frequently prescribed for sleep disorder management, their long-term usage is restricted due to their severe adverse side effects in memory impairment, next day hangover, dizziness, headache, fatigue, falls, gastrointestinal upset, tolerance, rebound worsening of sleep problems at discontinuation, and addiction.^3^ As a result, a considerable portion of the U.S. adult population are searching for a safe and efficacious alternative from natural sources. Nevertheless, natural products with sufficient scientific data in support of their usage for sleep and/or balanced mood are very scarce.

While there are some psychotropic clinical and anecdotal data available to support the usage of sedative and anxiolytic plants like kava kava, *Valeriana officinalis* (valerian), *Lavandula angustifolia* (lavender), *Matricaria recutita* (chamomile), wuling (mycelia of precious *Xylaria nigripes* (Kl.) Sacc), *Passiflora incarnata* (passionflower), *Humulus lupulus* (hops), and *Melissa officinalis* (lemon balm) as sleep aid supplements, potential hepatotoxicity, slow onset of effect, and inconsistent efficacy compared to placebo limit their widespread application, leaving synthetic melatonin as the primary sleep aid choice available on the market.^4,5^As a result, there still is an unfulfilled vacancy in the demand for a safe and efficacious dietary supplement from natural sources with clinically proven efficacy on the state of mood and sleep to be considered a natural alternative to melatonin.

Cortisol, a glucocorticoid released from the adrenal cortex, is more than a stress hormone known to regulate multiple vital metabolic and physiological functions in the body through the hypothalamic-pituitary-adrenal (HPA) axis. Sleep is among several physiologic functions regulated by HPA axis,^6,7^ where increased cortisol level and poor sleep quality have been correlated to dysregulated HPA axis. For example, daytime cortisol level and its correlation to sleep disturbance were assessed in healthy older subjects who frequently report declined sleep quality.^8^ A total of 672 older adults within the 67–90-year-old age group were included in this study. Regression analyses were conducted for a daytime cortisol level and sleep characteristics (fragmentation, wake after sleep onset, and duration), which were derived from wrist actigraphy. It was found that both higher fragmentation score and longer wake after sleep onset were significantly associated with higher daytime cortisol.

In a similar study, compared with healthy young subjects (average of 21 years old), healthy older adults (average of 71 years old) were found to sleep more poorly at night, as indicated by increased wake time and decreased percentage of slow-wave sleep. Similarly, interaction between altered HPA-axis functions and disturbed sleep was underlined by studies indicating a positive correlation between total wake time and 24-hour urinary cortisol secretion^9^ and increased plasma cortisol levels with the number of nocturnal awakenings.^10^ These reports support the hypothesis that poor sleep quality is closely linked to cortisol level, whereby, reduced cortisol level is a fundamental step for maintaining improved sleep quality by night and well-managed stress by day.

Previously, the mood-enhancing effect of UP165, a *Zea mays* (commonly known as corn) leaf extract standardized for 6-methoxybenzoxazolinone (6-MBOA) content has been reported.^11^ In this active comparator clinical trial, when UP165 at 250 mg once per day in the morning was orally administered to participants with mild depression and anxiety for 8 weeks, statistically significant improvements in mood states were observed. The potential sleep aid application of UP165 was also reported using a pentobarbital-induced *in vivo* sleep study, wherein UP165 produced comparable activity to that of melatonin in reducing sleep onset latency and increasing sleep duration of mice.^12^

These clinically meaningful desired outcomes were postulated partially as a result of the mechanisms of action of UP165 such as (1) structural similarity of the active in UP165 to that of melatonin and the production of a melatonin-like effect, (2) increased endogenous biosynthesis of serotonin and melatonin through induction of rate-limiting enzymes, such as tryptophan-5-hydroxylase, and *N*-acetyltransferase, respectively,^13,14^ (3) reduced or inhibited activity of the liver enzyme tryptophan dioxygenase and freeing up of more starting material for serotonin/melatonin conversion,^14^ and (4) skeletal muscle relaxation property of the active in UP165.^15^ Based on these preclinical facts, a double-blind, placebo-controlled clinical trial was designed to assess the hypothesis that participants supplemented with UP165 (compared to placebo) would exhibit improvements in mood state, night-time sleep time, sleep quality, and metabolic parameters associated with stress, such as cortisol.

## MATERIALS AND METHODS

### Receptor binding assay

To test the ability of UP165 to bind to the melatonin binding site of the melatonin receptor 1 (MT1) and 2 (MT2) melatonin receptors, membrane-bound human MT1 and MT2 melatonin receptors were incubated with UP165 in the presence of [^125^I] 2-Iodomelatonin, an agonist of both receptors. The ability of UP165 to compete against [^125^I] 2-Iodomelatonin for binding was demonstrated by inhibition of [^125^I] 2-Iodomelatonin binding to the melatonin receptors.

Cell membranes from human recombinant Chinese hamster ovary (CHO)-K1 cells expressing the human melatonin MT1 receptor were harvested and incubated with UP165 at concentrations 0.78, 1.56, 3.12, 6.25, 12.5, 25, 50, 100, 200, and 400 *μ*g/mL for MT1 and 3.12, 6.25, 12.5, 25, 50, 100, 200, 400, 800, and 1600 *μ*g/mL for MT2 for three hours at room temperature, along with 0.05 nM [^125^I] 2-Iodomelatonin radioligand. The membranes were filtered and washed, and the amount of [^125^I] 2-Iodomelatonin counted. The percent inhibition for the test compound was calculated compared to a 1% dimethylsulfoxide (DMSO) vehicle control. Greater than 50% inhibition was considered to have met the significance criteria.

### Clinical study design

This study enrolled 45 healthy volunteers (24 female and 21 male subjects) to participate in a 6-week sleep and overall well-being trial. Subjects were randomized to receive UP165 (Supplement) or a nonactive corn starch placebo (Placebo). Subjects (age range 19–73 years) were instructed to consume the supplement or placebo ∼60 minutes before bedtime daily for 4 weeks after a 2-week baseline period (no supplementation). The active test material, UP165, was administered orally at 250 and 500 mg/day. Following a 2-week baseline monitoring period, groups were assigned. Sleep Quality (latency, efficiency, and deep/rapid eye movement [REM] time) was monitored nightly, and POMS and Pittsburgh Sleep Quality Survey were monitored weekly for each subject in each group for a period of 4 weeks. Salivary cortisol level was also determined for all subjects on Day 0 and weekly for 4 weeks.

### Study measuring tools

(1) Objective sleep quality was measured using the Garmin Vivosmart^®^ 4 fitness activity tracker. The tracker includes advanced sleep monitoring with REM sleep, light sleep, deep sleep, and movement throughout the night. (2) Subjective sleep quality and efficiency: the Pittsburgh Sleep Quality Index (PSQI) is a well-validated self-rated questionnaire that assesses sleep quality and disturbances over a 4-week time interval. Nineteen individual items generate seven component scores such as subjective sleep quality, sleep latency, sleep duration, habitual sleep efficiency, sleep disturbances, use of sleeping medication, and daytime dysfunction.

The sum of scores for these seven components yields one global score, with higher scores corresponding to worsening sleep quality and efficiency.^16^ (3) Mental Well-Being & Psychological Mood State (POMS survey): the POMS is a well-validated psychological rating scale used to assess both overall well-being (Global Mood State) and distinct specific mood states (Subscales). The POMS measures six different dimensions of mood over a period of time, including Tension/Anxiety, Anger/Hostility, Vigor/Activity, Fatigue/Inertia, Depression/Dejection, and Confusion/Bewilderment. (4) Salivary Cortisol Level: cortisol is a major glucocorticosteroid hormone produced by the adrenal cortex, it is actively involved in multiple biological pathways. Salivary cortisol is unbound to protein and is highly correlated to serum cortisol.^17^

### Inclusion criteria

Healthy adults, able to provide informed consent, and with the ability/desire to participate in a 4-week supplementation regimen and sleep quality study were included in the study.

### Exclusion criteria

Inability to complete prescribed supplement regimen, current use of incompatible medications or supplements, high use of caffeine (500 mg/day) or other stimulants, off shift and night shift workers, and those diagnosed with severe sleep disorders were excluded from this study.

### Ethics and regulatory approval

The study was performed in compliance with the requirements of Good Clinical Practice/International Council for Harmonisation (GCP/ICH) Guidelines for clinical trials. The Study gained full board review from Institutional Review Board (IRB) on May 14, 2020, by WCG Aspire IRB. Protocol number Maizinol (UP165) 2020 IRB Tracking number 20201258.

### Participant informed consent

All subjects provided written informed consent to participate in the study before being screened and a copy was provided for their information. Subjects were also free to withdraw from the study at any time without giving a reason.

### Compliance

Adverse event (AE) data were collected by subject reporting of any AE, which were captured in subject charts. This study was conducted in a healthy population, and subjects were contacted weekly by phone call, text, or email. Subjects were given a number to call if there was any issue to report. Compliance was a combination of wearing the device to capture data, staying away from consumption of excluded supplements that could interfere with test materials activity, and adhering to scheduled daily supplement consumption, which was verified through pill count to ensure the investigational product was taken as per protocol. This was done during the weekly contact with the participants.

### Statistical analysis

Data were analyzed using John's Macintosh Project (JMP) 14.0 data analysis software (SAS Institute, Cary, NC, USA). The results are represented as mean ± standard deviation. Statistical significance between groups was calculated by means of single-factor analysis of variance (ANOVA) followed by a paired *t*-test. *P* values less than or equal to .05 (*P* ≤ .05) were considered statistically significant. Equal Variance Test was performed using the Brown-Forsythe test. When the normality test failed for nonparametric analysis in Shapiro–Wilk, data were subjected to Mann–Whitney sum ranks for *t*-test and Kruskal–Wallis one-way ANOVA on ranks for ANOVA. When the treatment group sizes were unequal, Dunn's test was used for pairwise comparisons and comparisons against placebo group following rank-based ANOVA. Percent changes from baseline were calculated as % changes = [(Mean value of post supplement − mean value of presupplement)/(Mean value of presupplement)] × 100.

## RESULTS

### Receptor binding assay result

**FIG. 1.**
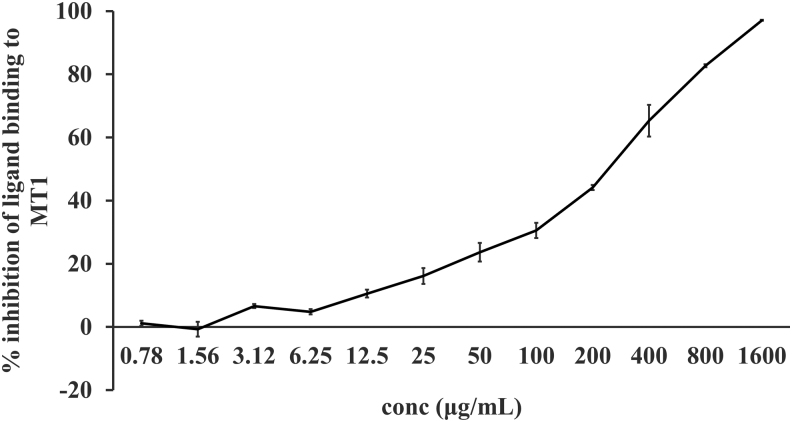
Percent inhibition of ligand binding to MT1. Human recombinant CHO-K1 cells expressing the human melatonin MT1 receptor were incubated with UP165 at 10 concentrations and run in duplicate. The percent inhibition for the test compound was calculated compared to a 1% DMSO vehicle control. CHO, Chinese Hamster Ovary; DMSO, dimethylsulfoxide; MT1, melatonin receptor 1.

**FIG. 2.**
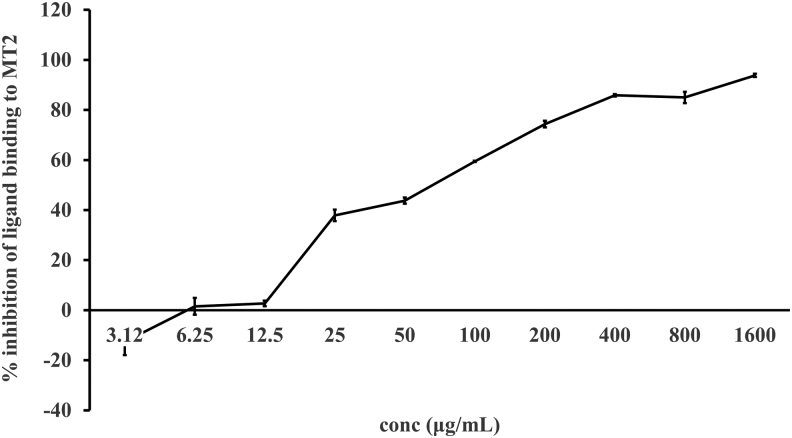
Percent inhibition of ligand binding to MT1. Human recombinant CHO-K1 cells expressing the human melatonin MT2 receptor were incubated with UP165 at 10 concentrations and run in duplicate. The percent inhibition for the test compound was calculated compared to a 1% DMSO vehicle control. MT2, melatonin receptor 2.

UP165 (Lot. No. FP041019-01) was tested in duplicate at 10 concentrations for its MT1 and MT2 binding affinity. UP165 showed a fourfold increase in MT2 receptor binding affinity. UP165 had a dose-responsive curve with an IC_50_ of 229 *μ*g/mL and an inhibition constant (*K_i_*) of 119 *μ*g/mL for MT1 receptor and an IC_50_ of 56.6 *μ*g/mL with inhibition constant *K_i_*: 28.3 *μ*g/mL for MT2 (Figs. 1 and 2).

### Clinical study compliance and completion rate

Participants were compliant for all the procedures and supplementation for the duration of the study. Forty-two (23 female and 19 male) participants completed the study. No AE was reported for test article or placebo. Three participants dropped out of study due to scheduling conflicts (Table 1).

**Table 1.** Study Enrollment and Dropout

Study groups	Enrolled (F = 24; M = 21)	Completed subjects (F = 23; M = 19)
A: 250 mg of UP165	*N* = 15	*N* = 14 (7F, 7M)
B: 500 mg of UP165	*N* = 15	*N* = 13 (8F, 5M)
C: Placebo group	*N* = 15	*N* = 15 (8F, 7M)
Total	45	42

The study enrolled 45 volunteers to participate in a 6-week trial.

### Effect of UP165 on deep sleep

Increase in the deep sleep time was observed for participants who were supplemented with the test article, UP165. Compared to placebo, these increases in the deep sleep time were statistically significant for participants supplemented with the 250 mg/day UP165 at week 2 and 3 with *P* values .011 and .002, respectively (Table 2). Similarly, the 500 mg/day UP165 group showed a statistically significant increase in the deep sleep time at weeks 2 and 3 with *P* values .0098 and .0081, respectively, when compared to placebo.

**Table 2.** Effect of UP165 on the Average Deep Sleep Values on Healthy Participants

Group	*N*	Deep sleep (mean ± SD)				
		Baseline	Week 1	Week 2	Week 3	Week 4
UP165 (250 mg/day)	12	3825.9 ± 1653.3	5571.9 ± 3340.1 (*P* = .123)	5154.5 ± 2457.5 (*P* = .011)	4992.75 ± 2184.8 (*P* = .002)	5518.5 ± 2259.8 (*P* = .055)
UP165 (500 mg/day)	13	4055.5 ± 2058.8	4506.5 ± 2083.4 (*P* = .475)	4916.2 ± 2067.4 (*P* = .0098)	5125.1 ± 2965.9 (*P* = .0081)	5665.3 ± 3099.3 (*P* = .074)
Placebo	14	3473.9 ± 1644.1	4019.0 ± 1358.3	3002.6 ± 1460.8	2710.9 ± 996.9	3740.9 ± 2226.3

The study enrolled 45 volunteers to participate in a 6-week trial. Participants (age range 19–73) consumed the supplement or placebo ∼60 minutes before bedtime daily for 4 weeks after a 2-week baseline period (no supplementation). Data in parenthesis are *P* values against placebo for each treatment group. All measurements are in seconds.

SD, standard deviation.

**FIG. 3.**
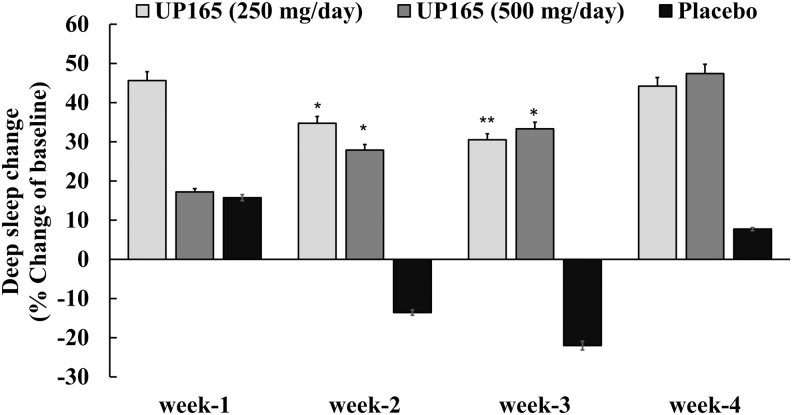
Percent baseline change of UP165 on deep sleep of healthy participants. The study enrolled 45 volunteers to participate in a 6-week trial. Participants (age range 19–73) consumed the supplement or placebo ∼60 minutes before bedtime daily for 4 weeks after a 2-week baseline period (no supplementation). The active test material UP165 was administered orally at 250 and 500 mg/day. *P < .05; **P ≤ .001 versus placebo.

When the percent of increase from the baseline was computed, ranges of 30.5–45.6% and 27.9–47.4% increase in deep sleep time were observed for those participants supplemented with 250 and 500 mg/day UP165, respectively (Fig. 3). In contrast, subjects in the placebo group showed a decrease in the deep sleep time in weeks 2 and 3 with a slight increase at week 4 with 7.7%. At the end of the study, the increase in the deep sleep time for the participants showed trends with *P* values .062 and .055 for the 250 and 500 mg/day, respectively, when compared to the placebo group. These objective measures clearly showed that UP165 is a dietary supplement with marked impact on sleep quality as reflected by statistically significant increased deep sleep time at those indicated time points.

### Effect of UP165 on total sleep time

**FIG. 4.**
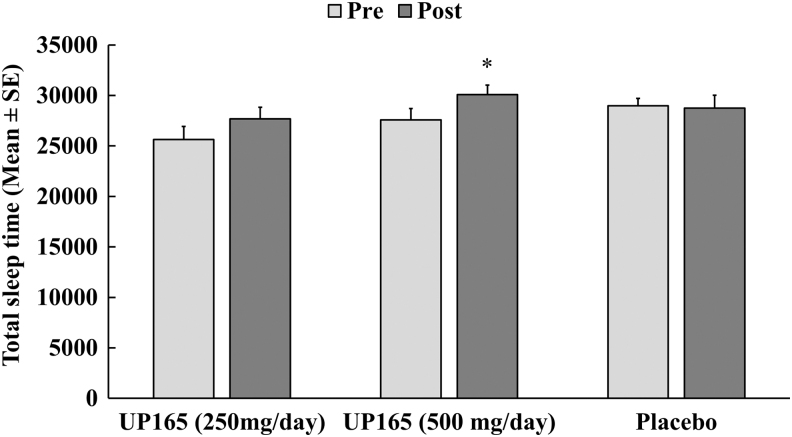
The average total sleep time before and after three supplements. The study enrolled 45 volunteers to participate in a 6-week trial. Participants (age range 19–73) consumed the supplement or placebo ∼60 minutes before bedtime daily for 4 weeks after a 2-week baseline period (no supplementation). The active test material UP165 was administered orally at 250 and 500 mg/day. Data represented as mean ± SE. *P < .05 versus baseline. SE, standard error.

Compared to the baseline, UP165 resulted in a moderate, but statistically significant (*P* ≤ .05), increase in total sleep time when supplemented to healthy subjects at a dosage level of 500 mg/day (Fig. 4). At the end of the 4-week study period, there were 8.0% and 9.1% increases in sleep time for the 250 and 500 mg/day UP165 groups, respectively. Total sleep time was unaffected for the placebo group. These data further indicate that the significant impact of UP165 supplementation on healthy subjects is on sleep quality and efficiency with moderate improvement in sleep quantity.

### Effect of UP165 supplementation on sleep quality improvement as it was measured by PSQI

**FIG. 5.**
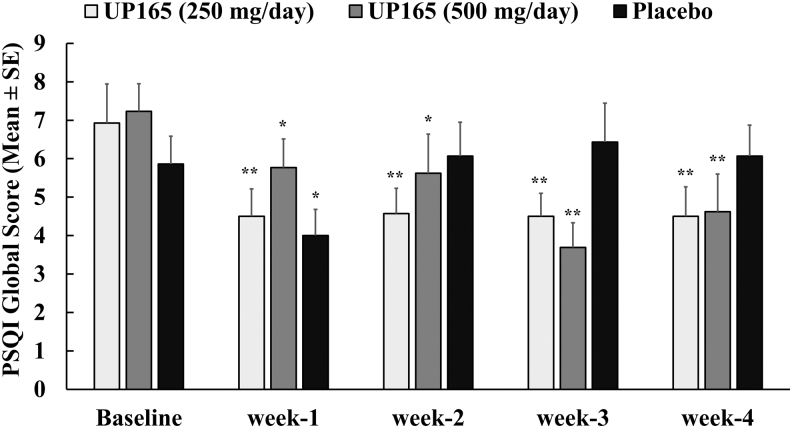
PSQI global scores for three supplement groups. The study enrolled 45 volunteers to participate in a 6-week trial. Participants (age range 19–73) consumed the supplement or placebo ∼60 minutes before bedtime daily for 4 weeks after a 2-week baseline period (no supplementation). The active test material UP165 was administered orally at 250 and 500 mg/day. Data represented as mean ± SE. *P < .05; **P ≤ .001 versus baseline. PSQI, Pittsburgh Sleep Quality Index.

The PSQI is a self-rated questionnaire that assesses sleep quality and disturbances over a 4-week time interval. Participants who were supplemented with UP165 showed a statistically significant improvement in self-rated questionnaires, indicating the effect of UP165 in enhancing sleep quality and efficiency in healthy participants. UP165 supplementation produced significantly improved sleep quality as early as week 1 (*P* ≤ .5) and its effect was sustained throughout the 4-week duration (Fig. 5). As seen in Figure 5 and Table 3, during this supplementation period, UP165 caused improvement in sleep quality as much as 48.9% and 35.1% from baseline when it was administered at 500 and 250 mg/day, respectively.

**Table 3.** Effect of UP165 Supplementation on Sleep Quality Improvement as Measured by Pittsburgh Sleep Quality Index

Group	*N*	PSQI Global Score (percent difference of baseline) (one-way ANOVA vs. placebo,* P*-value)			
		Week 1	Week 2	Week 3	Week 4
UP165 (250 mg/day)	14	−35.1% (*P* = .924)	−34.0% (0.072)	−35.1% (0.197)	−35.1% (0.041)
UP165 (500 mg/day)	13	−20.2% (*P* = .344)	−22.3% (*P* = .285)	−48.9% (0.055)	−36.2% (0.037)
Placebo	14	−31.6%	3.6%	9.7%	3.6%

The study enrolled 45 volunteers to participate in a 6-week trial. Participants (age range 19–73) consumed the supplement or placebo ∼60 minutes before bedtime daily for 4 weeks after a 2-week baseline period (no supplementation).

ANOVA, analysis of variance; PSQI, Pittsburgh Sleep Quality Index.

The placebo effect (31.6% increase in PSQI from baseline) observed in week 1 for the placebo group was nonexistent for the remainder of the study period (Table 3). After 4 weeks of daily supplementation, it was found that, participants in the UP165 group showed a 35.1% and 36.2% improvement in quality of sleep when UP165 was administered at 250 and 500 mg/day, respectively. These improvements observed after 4 weeks of daily supplementation of UP165 were statistically significant for both the dosages compared to placebo (*P* = .041 for placebo vs. 250 mg/day UP165 and *P* = .037 for placebo vs. 500 mg/day UP165). On the other hand, the change in improvement for the placebo group was only 3.7% (Fig. 5 and Table 3). These PSQI data are a confirmation that UP165 supplementation indeed has a significant impact on sleep quality and efficiency.

### Effect of UP165 on salivary cortisol level

**FIG. 6.**
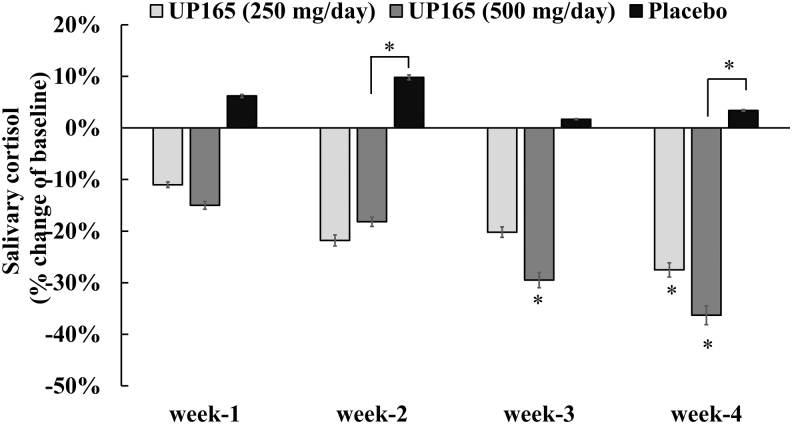
Percentage changes of salivary cortisol levels from supplements of three test articles. The study enrolled 45 volunteers to participate in a 6-week trial. Participants (age range 19–73) consumed the supplement or placebo ∼60 minutes before bedtime daily for 4 weeks after a 2-week baseline period (no supplementation). The active test material UP165 was administered orally at 250 and 500 mg/day. *P < .05 versus baseline or placebo.

Cortisol, one of the major glucocorticoid hormones secreted by the adrenal cortex, is among the hormones that regulate human sleep, with its increase being associated with poor sleep quality. In this clinical study, subjects supplemented with UP165 showed a progressive decrease in salivary cortisol level through the course of the study (Fig. 6 and Table 4). A statistically significant decrease in cortisol level was observed for the 500 mg/day group of subjects at week 3 (*P* = .045) and week 4 (*P* = .006) compared to baseline. Subjects in the 250 mg/day group showed a statistically significant reduction in cortisol level on week 4 compared to baseline (*P* = .04). The decrease in cortisol level was also statistically significant against placebo at week 2 and 4 (*P* = .023) for subjects who were supplemented with UP165 at 500 mg/day.

**Table 4.** Effect of UP165 on Salivary Cortisol Level

Group	*N*	Cortisol pg/mL (mean ± SD)				
		Baseline	Week 1	Week 2	Week 3	Week 4
UP165 (250 mg/day)	14	3529 ± 1686	3139 ± 1670 (*P* = .581)	2759 ± 940 (*P* = .147)	2815 ± 1838 (*P* = .141)	2557 ± 972 (*P* = .040)
UP165 (500 mg/day)	13	2920 ± 1008	2482 ± 1189 (*P* = .144)	2389 ± 1263 (*P* = .069)	2059 ± 1067 (*P* = .045)	1861 ± 749 (*P* = .006)
Placebo	14	3294 ± 1092	3498 ± 2916 (*P* = .709)	3616 ± 2063 (*P* = .587)	3351 ± 2288 (*P* = .921)	3406 ± 2365 (*P* = .864)

The study enrolled 45 volunteers to participate in a 6-week trial. Participants (age range 19–73) consumed the supplement or placebo ∼60 minutes before bedtime daily for 4 weeks after a 2-week baseline period (no supplementation). Data in parenthesis are *P* values against baseline for each treatment group.

Interestingly, as shown in Figure 6, while the 250 and 500 mg/day UP165-supplemented subjects experienced an 11% and 15% reduction in cortisol level in week 1, respectively, in the same time frame, the placebo group showed a 6.2% increase in cortisol level. These patterns of cortisol changes were sustained for the duration of the study. Consistently, while participants in the UP165 group showed a reduction in cortisol level, the placebo participants showed a mild to moderate increase in cortisol level, week after week, throughout the study. These reductions in cortisol level were dose correlated for the UP165 group.

Reductions of cortisol level such as 11.0%, 21.8%, 20.2%, and 27.5% for the 250 mg/day and 15.0%, 18.2%, 29.5%, and 36.3% for the 500 mg/day UP165 were observed for weeks 1–4, respectively. In contrast, 6.2%, 9.8%, 1.7%, and 3.4% increase in the salivary cortisol level was observed for the placebo group for the same time frame. These cortisol findings align well with the deep sleep findings described above, affirming the improved sleep quality and efficiency experienced as a result of UP165 supplementation.

### Effect of UP165 on POMS

**FIG. 7.**
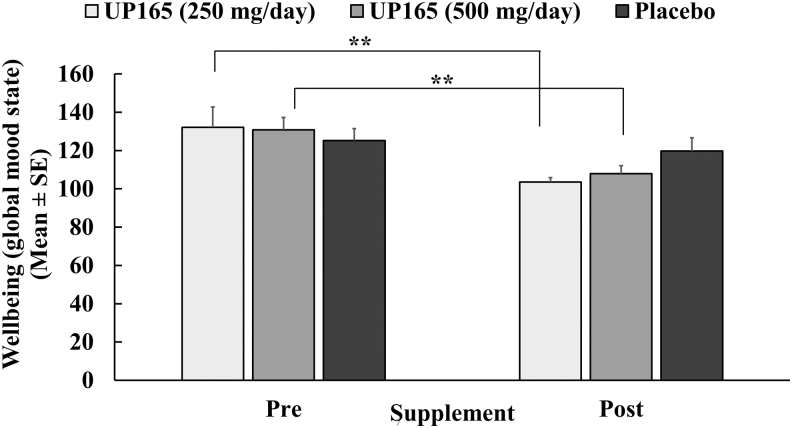
Global mood state measurements from three test articles. The study enrolled 45 volunteers to participate in a 6-week trial. Participants (age range 19–73) consumed the supplement or placebo ∼60 minutes before bedtime daily for 4 weeks after a 2-week baseline period (no supplementation). The active test material UP165 was administered orally at 250 and 500 mg/day. **P ≤ .001 versus baseline.

The POMS is a well-validated psychological rating scale used to assess both overall well-being (Global Mood State) and distinct specific mood states (Subscales). Data were analyzed to determine the presupplementation and postsupplementation effects of the test article, UP165, on the overall well-being of test subjects (Fig. 7). A dose-correlated improvement in overall well-being, as determined by a “Global Mood State,” was observed for subjects supplemented with the active test material, UP165. A statistically significant improvement in overall well-being was observed for the subjects who were supplemented with the test material, UP165, for both the dosages (*P* ≤ .001). There were 22% and 17% improvements in overall well-being for the 250 and 500 mg/day subjects, respectively, when compared to the presupplementation of test articles. Subjects in the placebo group showed a 4% improvement in overall well-being postsupplementation (Fig. 7).

The psychological mood state profile of subjects was also assessed for each subject in the supplementation groups for each dimension of mood state. As seen in Table 5, subjects in the UP165 groups showed statistically significant improvements in subscales of POMS such as depression, tension, fatigue, and confusion at both dosages. No statistical significance on vigor and anger for the 500 mg/day and on vigor for the 250 mg/day was observed for the active test material. There were 58% and 37% decreases in depression and 36% and 36% reductions in the anxiety/tension for subjects who were supplemented with 250 and 500 mg/day UP165, respectively. Improvement changes for the placebo-supplemented group were very minimal for all the parameters analyzed.

**Table 5.** Profile of Mood States Improvements from Baseline

Group	*N*	Psychological Mood State Subscales (POMS) (% difference from baseline)					
		Vigor	Anger	Confusion	Fatigue	Depression	Tension
UP165 (250 mg/day)	14	+2% (*P* = .7698)	−37% (*P* = .0004)	−40% (0.0059)	−43% (*P* = .0032)	−58% (*P* = .0036)	−37% (*P* = .0013)
UP165 (500 mg/day)	13	+5% (*P* = .4957)	−23% (*P* = .0770)	−38% (*P* = .0079)	−42% (*P* = .0040)	−36% (*P* = .0480)	−36% (*P* = .0115)
Placebo	15	−2% (*P* = .5847)	−19% (*P* = .1933)	−15% (*P* = .0548)	−9% (*P* = .4210)	−14% (*P* = .1971)	−14% (*P* = .0637)

The study enrolled 45 volunteers to participate in a 6-week trial. Participants (age range 19–73) consumed the supplement or placebo ∼60 minutes before bedtime daily for 4 weeks after a 2-week baseline period (no supplementation). Data in parenthesis are *P* values against baseline for each treatment group.

POMS, Profile of Mood States.

## DISCUSSION

Despite significant research efforts for sleep and mental health management, to date, available intervention options from natural sources are very limited. In particular, the sleep aid market is significantly saturated by high doses of synthetic melatonin or cocktails of melatonin and other botanical extracts with minimal to no clinical evidence to support their meaningful clinical usage. Higher dosages of synthetic melatonin supplements could also produce side effects such as morning grogginess, daytime sleepiness, headache, dizziness, and hypothermia.^18–20^

Supplementation of a standardized corn leaf extract, UP165, improved sleep quality of clinical study participants by increasing the deep sleep stage of sleep approximately by half an hour. The total deep sleep times at the start of the study period for the 250 mg/day, 500 mg/day, and placebo were 64, 68, and 58 minutes, respectively, indicating neither the supplement nor the placebo group was to the level considered good-quality sleep per night. Following the 4-week supplementation, the deep sleep times were found to be increased to 92, 94, and 62 minutes for the 250 mg/day, 500 mg/day, and placebo group, respectively. Such increase of deep sleep time for high-dose Maizinol group was statistically significant at week 2 and 4 with *P* value at .025 and .014 compared to the baseline, while the low-dose group has a strong trend with *P* value at .051 at week 4.

Due to small number of subjects in each group of this pilot study, both low- and high-dose Maizinol group showed statistically strong trend of increased deep sleep time with *P* value at .062 and .055, respectively, compared to placebo group at the end of the study. However, the increase in deep sleep time among all three groups was found statistically significant (*P* = .0318) by analyzing covariance with a repeated measures model with week as the repeated variable and subject (denoted by ID) as the repeated experimental unit. It is believed that to have good-quality sleep, one has to have a minimum of 90 minutes total deep sleep time per night.^21–23^ These data clearly show that, supplementation of UP165 helped the participants to achieve a good quality of sleep per night by increasing the deep sleep stage of sleep.

The increase in the deep sleep time observed in the UP165-supplemented group was possibly a direct reflection of the fourfold higher affinity of UP165 to the MT2 receptor, which is known to induce deep sleep when activated. The improved sleep quality findings from the sleep tracker were also verified by the PSQI questionnaire with participants showing a 10-fold increase in quality and efficiency of sleep improvement as a result of 250 mg/day of UP165, compared to that of participants who were supplemented with the placebo.

Again, substantiating the objective measures from the individual sleep tracker, when participants were asked about their mental health status using the POMS questionnaire, they provided statistically significant improvements in mood state and well-being compared to their baseline (37–58% improvement at 250 mg/day and 36–42% improvement at 500 mg/day), whereas the improvements for the placebo group were very minimal (9–15% improvement) and statistically nonsignificant.

The clinical superiority in performance observed for the UP165-supplemented participants compared to the placebo was closely correlated to the level of salivary cortisol during the course of the study. It has been known that high cortisol level is strongly linked to premature nocturnal awakening, decrease in deep sleep, and hence poor sleep quality.^24,25^ Reducing cortisol level is a fundamental step for maintaining sleep homeostasis and, hence, improved sleep quality. In these clinical trial data, there was an inverse correlation between deep sleep and cortisol level. These data highlight the importance of reducing cortisol level for improved sleep quality and efficiency, and participants who were supplemented with UP165 showed significantly improved sleep quality and efficiency as a result.

It has been well documented that sleep displays a close and inverse relationship with increased level of cortisol and HPA axis regulation.^25^ For instance, in a clinical setting, when 33 healthy young men were subjected to partial or total sleep deprivation, there were statistically significant increases in plasma cortisol levels the next day for both scenarios, suggesting that even partial acute sleep loss delays the recovery of the HPA from early morning circadian stimulation. Based on these findings, the authors concluded that the increase in plasma cortisol level and delayed HPA response could likely involve an alteration in negative glucocorticoid feedback regulation.^24^ Association of these two factors (inverse correlation of cortisol level and sleep quality) was observed in our clinical trial, which produced reduced salivary cortisol level and improved slow-wave deep sleep in the UP165 treatment groups, which could be translated as better sleep quality.

These clinical study results support the fact that participants who were supplemented with a standardized corn leaf extract, UP165, had significantly reduced cortisol levels (up to 36%) and increased slow-wave deep sleep time (up to 30 minutes), which translated to better sleep quality when compared to the placebo group. These clinical findings also added additional dimension to the mechanism of action of UP165, in that, by reducing cortisol level, there is a possibility that UP165 could correct a dysregulated HPA axis, leading to normal homeostasis of vital physiological functions, including sleep. This clinical evidence in association with the ability of UP165 to induce the body to synthesize endogenous melatonin, directly binding to MT1 and MT2 receptors, being structurally similar to melatonin, and having a muscle relaxation property, could produce a potential additive and/or synergistic effect that will lead to enhanced induction and maintenance of sleep.

Collectively, UP165 showed a statistically significant (1) dose-correlated reduction in salivary cortisol level compared to placebo; (2) improved deep sleep time in a dose-correlated manner; (3) increased total sleep time compared to baseline; (4) improved sleep quality and efficiency as measured by PSQI; and (5) improved overall well-being as measured by POMS scale. Therefore, UP165 could be used as a safe nutritional supplement for a 24-hour support for a better quality and efficiency of sleep at night and for improved mood state and overall well-being by day. These data suggest the possibility of the standardized, natural, hormone-free, corn leaf extract, UP165, to be considered a natural alternative to a synthetic melatonin.
